# Mapping genome-wide transcription factor binding sites in frozen tissues

**DOI:** 10.1186/1756-8935-6-30

**Published:** 2013-09-16

**Authors:** Daniel Savic, Jason Gertz, Preti Jain, Gregory M Cooper, Richard M Myers

**Affiliations:** 1 HudsonAlpha Institute for Biotechnology, 601 Genome Way, Huntsville, AL 35806, USA

**Keywords:** ChIP-seq, Frozen tissues, Gene regulation, Genomics, Tissue specificity

## Abstract

**Background:**

Genome-wide maps of transcription factor binding sites in primary tissues can expand our understanding of genome function, transcriptional regulation, and genetic alterations that contribute to disease risk. However, almost all genome-wide studies of transcription factors have been in cell lines, and performing these experiments in tissues has been technically challenging and limited in throughput.

**Results:**

Here we outline a simple strategy for mapping transcription factor binding sites in frozen tissues that utilizes dry pulverization of samples and is scalable for high-throughput analyses. We show that the method leads to accurate and reproducible chromatin immunoprecipitation next-generation sequencing (ChIP-seq) data, and is highly sensitive, identifying high-quality transcription factor binding sites from chromatin corresponding to only 5 mg of liver tissue.

**Conclusions:**

The enhanced reproducibility, robustness, and sensitivity of the dry pulverization method, in addition to the ease of implementation and scalability, makes ChIP-seq in primary tissues a widely accessible assay.

## Background

The assembly of a reference human genome coupled to the development of novel array and next-generation sequencing technologies has ushered in an era of high-resolution approaches for mapping functional genomic features [1-4], and higher-order nuclear architecture [5,6]. In particular, cataloging genome-wide binding profiles of diverse DNA-binding proteins through next-generation sequencing of chromatin immunoprecipitated DNA, or chromatin immunoprecipitation next-generation sequencing (ChIP-seq) [7,8], has deepened our understanding of transcription factor involvement in gene regulation. Although cell lines have played a seminal role in describing how transcription factors interact with the genome [1,2,9-13], transcription factor characterization in primary tissues is a critical next step. While cell models allow for a focused and controlled biological system that can be manipulated in a variety of ways, the use of cancer-derived or transformed cells [14], as well as artificial culture conditions, can make direct biological inferences about normal *in vivo* states challenging. Despite being more complex and heterogeneous, tissues are derived from an *in vivo* context that is subject to physiological conditions, and therefore the direct analysis of primary tissues should provide more relevant insights into endogenous biological functions. Indeed, several influential ChIP-seq studies have been successfully performed in diverse mouse tissues [15-18], as well as normal [19] and diseased [20] human tissue samples. These investigations have illustrated the power of genomic assays in primary tissues for characterizing basic cellular functions and the genomic hallmarks common to disease states.

However, a major barrier to the broader utilization of ChIP-seq in tissues is that previously published strategies are much more technically challenging and labor intensive than similar protocols in cell lines. These tissue approaches have primarily relied on a mincing technique, wherein adult tissues are diced with a razor prior to fixation and homogenized in a Dounce homogenizer after fixation [21,22]. This dicing method is technically difficult and time consuming, making it less tractable for large-scale projects. The mincing approach can also lead to substantial sample loss through excessive handling of the tissue, while cross-contamination concerns diminish throughput. Importantly, differences in minced tissue sizes within and across samples can lead to heterogeneous fixation, which could limit reproducibility, assay sensitivity and overall data quality. This aspect may be particularly problematic for rare or scarce tissue samples. To circumvent the outstanding challenges with available methods, here we outline a ChIP-seq protocol for frozen tissue analyses that is simple, efficient, and involves minimal effort, making it easily implementable and amenable to higher throughput. We find the method to be robust across diverse tissue types and highly reproducible for both a general transcription factor (Rnap2) and sequence-specific factors (Ctcf and Rxrα). We also determined that our strategy is sensitive, accurately and reproducibly identifying binding sites with chromatin amounts that corresponded to only 5 mg of mouse liver (≈675,000 hepatocytes), far lower than previous protocols demand for liver tissue [21]. The approach also captures tissue specificity while further recapitulating results from previous genomic analyses.

## Results

### ChIP-seq in mouse tissues

**Figure 1:**
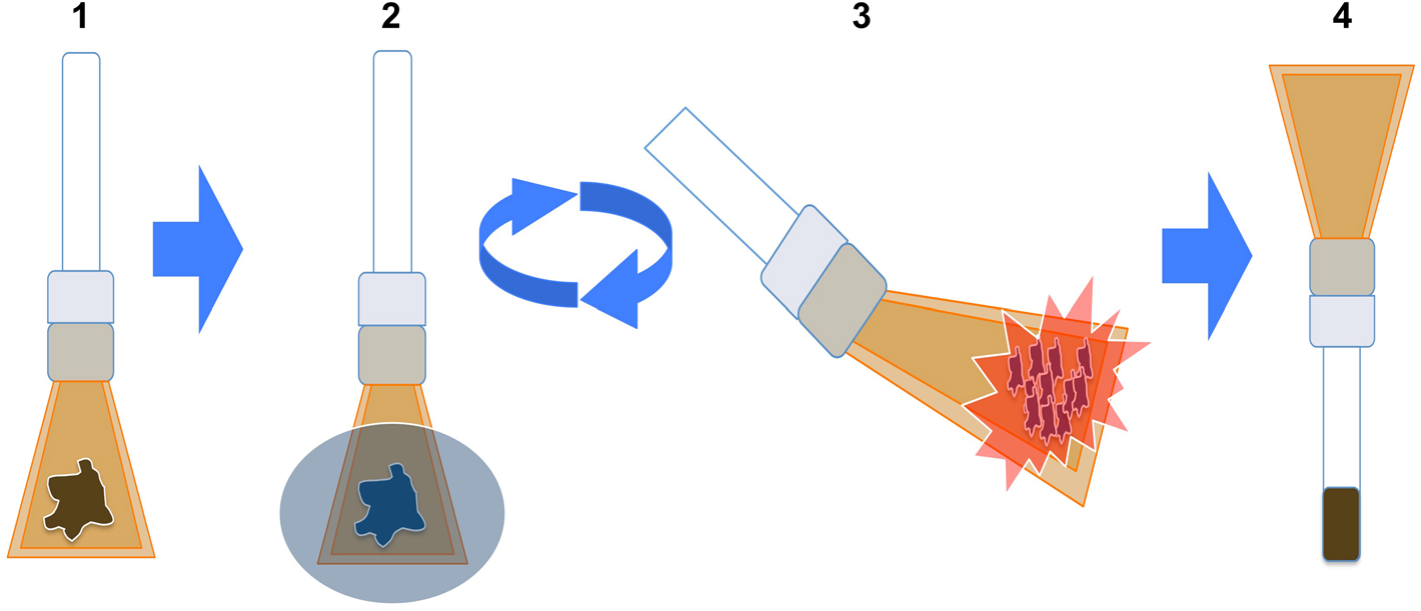
**Dry pulverization method overview.** Tissue is placed in Covaris tissueTUBES with adapters and attached glass vials (step 1). To keep samples both cold and brittle to facilitate pulverization, tissues are briefly submerged in liquid nitrogen (step 2) between successive rounds of pulverization (step 3). Following the pulverization of samples to a powder, the tissueTUBEs are inverted and tissue powder is collected into attached Covaris glass vials (step 4). Tissue powder is fixed, washed and stored as a pellet at −80°C.

To perform ChIP-seq in frozen tissues while minimizing tissue handling and cross-contamination concerns, we developed an approach that uses dry pulverization (Figure 1 and Methods). Tissues are placed in bags that can withstand high force and low temperatures and are pulverized on dry ice while repeatedly being cooled with liquid nitrogen. The repeated freezing with liquid nitrogen ensures that the tissue remains both frozen and brittle, facilitating the pulverization process. The resulting tissue powder is fixed with formaldehyde at room temperature and subsequent ChIP experimentation is performed in a manner identical to that in cell lines [23] (see Methods). Overall, the dry pulverization frozen tissue ChIP-seq protocol is simple and requires minimal steps, time and effort, making it easily implementable and scalable to higher throughput analyses. Supporting the enhanced throughput of the method, all murine samples were fixed in large batches that consisted of several distinct tissue sample types, and took approximately one hour to pulverize and fix.

To evaluate the dry pulverization technique, we tested mouse liver tissue with antibodies that target RNA polymerase II (Rnap2), the insulator CCCTC-binding factor (Ctcf) and the Retinoid X receptor α (Rxrα) nuclear receptor. We chose these factors to determine the feasibility of our strategy in defining genome-wide maps of distinct classes of DNA-binding proteins that are widely expressed in different tissue types, including RNA polymerases, canonical transcription factors, and proteins involved in maintaining genome structure.

**Figure 2:**
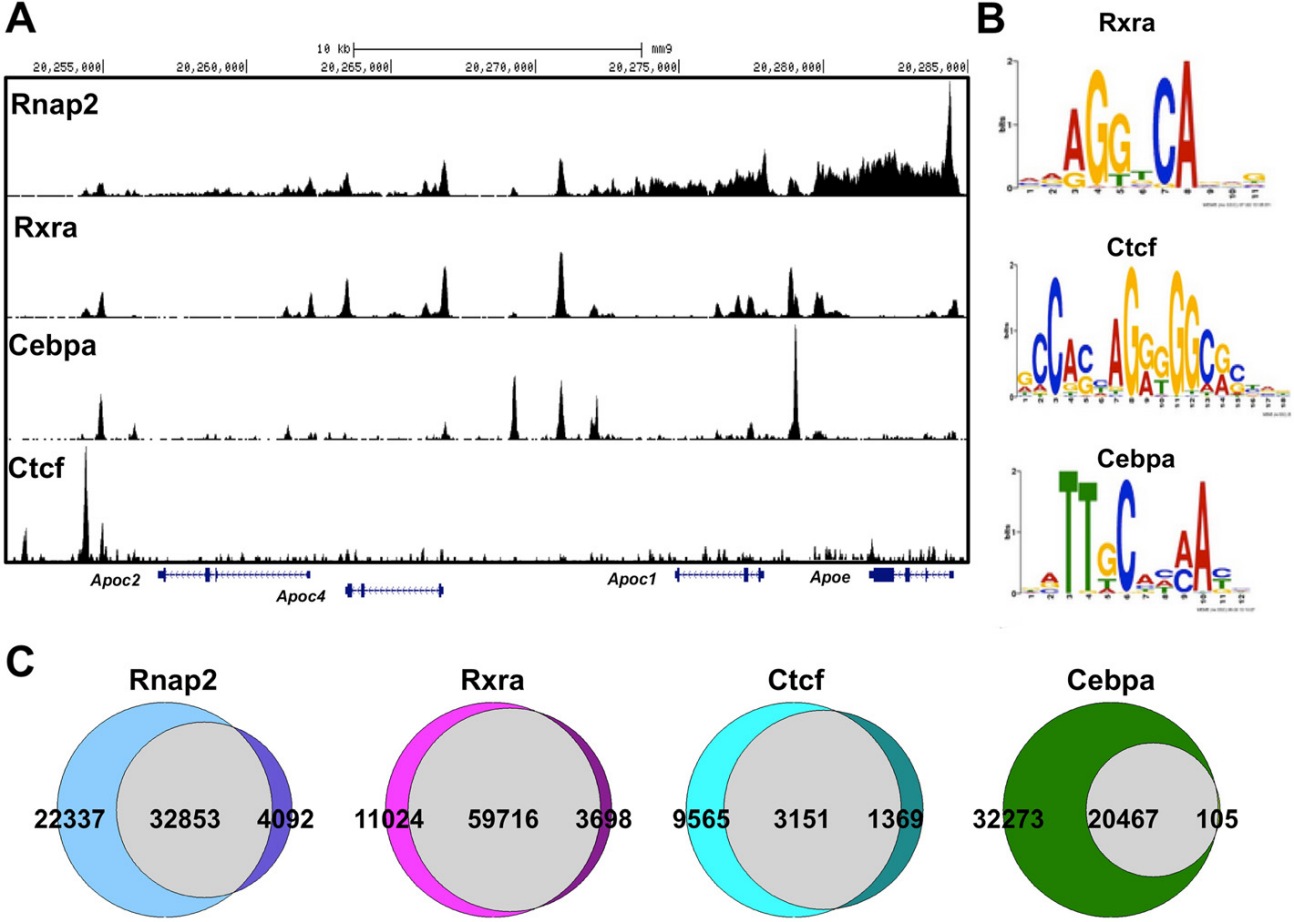
**ChIP-seq in murine liver. (A)** ChIP raw sequencing read enrichments for RNA polymerase II (Rnap2), Retinoid X receptor α (Rxrα), CCAAT/enhancer-binding protein α (Cebpα), and the CCCTC-binding factor (Ctcf) at an apolipoprotein cluster on mouse chromosome 7. **(B)** Images of the canonical motifs identified by multiple expectation maximization for motif elicitation (MEME) in liver. **(C)** Venn diagrams illustrate the degree of shared binding sites between liver biological replicates.

Using a common set of parameters for pulverization, fixation, and ChIP for all experiments (see Methods), we identified thousands of binding sites for Rnap2, Ctcf, and Rxrα in mouse liver (Figure 2A and Additional file 1: Table S1). For the sequence-specific factors (Ctcf and Rxrα), we determined that binding sites were enriched for their known canonical binding motifs (Figure 2B). We also performed ChIP analyses with independent biological replicates for each protein and found that more than 87% of transcription factor binding site overlap between replicates, illustrating the high reproducibility of the method (Figure 2C and Additional file 1: Table S1). As an additional validation of the technique, we performed ChIP-seq to identify CCAAT/enhancer-binding protein α (Cebpα) transcription factor binding sites, because this protein has an integral role in hepatic energy metabolism [24], and its binding pattern has been previously mapped in mouse livers [25], allowing for a direct comparison. Consistently with the results obtained from the other factors, we identified highly reproducible Cebpα binding sites enriched for the canonical motif (Figure 2B and Additional file 1: Table S1). We also evaluated the quality of our sequencing libraries by calculating library complexities, or the fraction of aligned sequences that map to a unique genome location compared with a randomly selected set of 10 million aligned sequences. For all ChIP experiments, including replicates, we found that our sequencing libraries harbored a high degree of complexity (Additional file 1: Table S1).

Given the success of the dry pulverization ChIP-seq protocol in liver, we assessed the performance of the assay in mouse brain, small intestine, and skeletal muscle samples with Rnap2, Ctcf, and Rxrα antibodies. The dry pulverization approach, with identical parameters for pulverization, fixation and ChIP, produced high-quality ChIP-seq data for each factor across all tissues despite their distinct histology (Additional file 1: Table S1), highlighting the robustness of the technique. For sequence-specific factors, the sites we identified were enriched for their canonical binding motifs (Additional file 2: Figure S1). Like our observations in liver, our results on binding sites were highly reproducible, with more than 75% of binding sites being shared between all pairs of biological replicates (Additional file 1: Table S1). We also found a high sequence complexity for all sequencing libraries (Additional file 1: Table S1). Collectively, these data indicate that the dry pulverization ChIP-seq method is robust across distinct tissue types and can reproducibly identify binding sites for distinct classes of DNA-binding proteins.

### Tissue specificity of genome-wide binding sites

**Figure 3:**
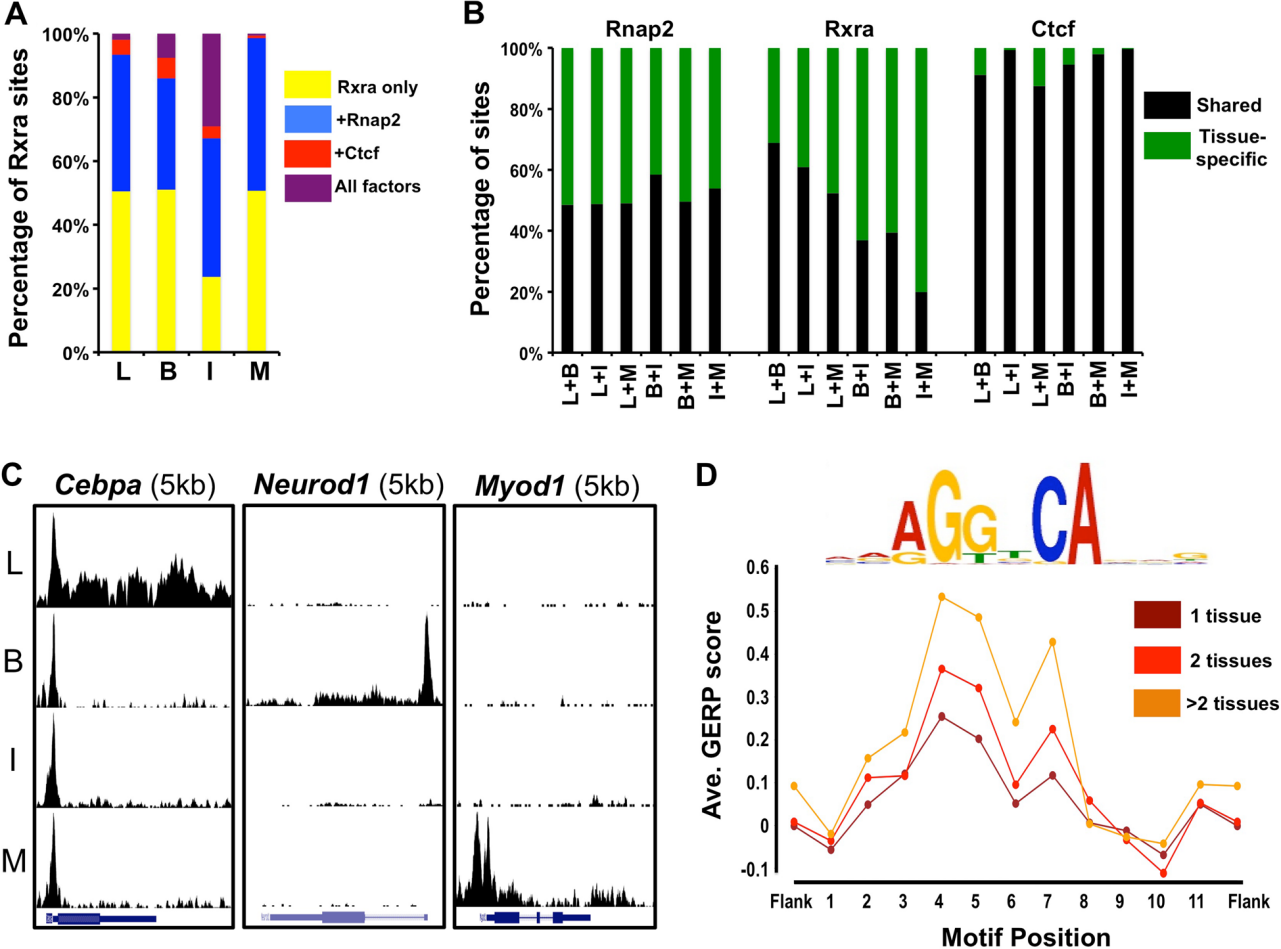
**ChIP-seq analyses across diverse murine tissues. (A)** Percentage of Rxrα binding sites shared with Rnap2 (blue), Ctcf (red) or both Rnap2 and Ctcf (purple) in liver (L), brain (B), small intestine (I) and skeletal muscle (M). Rxrα binding sites that do not colocalize with Rnap2 and Ctcf are shown in yellow. **(B)** Analysis of shared binding sites for Rnap2, Rxrα, and Ctcf between all pairwise tissue comparisons. The two tissues utilized for each comparison are given on the *x* axis. Shared binding sites are shown in black while tissue-specific sites are in green. **(C)** ChIP-seq raw sequencing read enrichments for Rnap2 at distinct genes illustrate tissue specificity of gene expression. Gene names and window sizes are given above. **(D)** Canonical motif genomic evolutionary rate profiling (GERP) scores at tissue-specific binding sites (dark red) and binding sites shared by two (red) or more (orange) tissues. The corresponding motif sequence is shown above the graph. GERP scores are significantly higher within bound Rxrα motifs relative to positions that are not within a motif but are within 250 bp of a binding site summit (*P* < 2.2 × 10^-16^, one-sided *t* test); further, there is a highly significant correlation between GERP score and position-specific motif dependencies on a particular nucleotide, with less degenerate positions being more highly conserved (*P* < 2.2 × 10^-16^, simple linear regression between GERP scores and the maximum individual nucleotide score at each position in the Rxrα motif position-specific weight matrix).

To assess whether the dry pulverization approach can capture meaningful biological information, we assessed the functional relevance of the binding data through intratissue and intertissue analyses. We evaluated the extent of binding colocalization between the three predominant DNA-binding proteins that we tested (Rnap2, Ctcf, and Rxrα) within each tissue (Figure 3A). We found that a higher proportion of Rxrα binding sites are shared with Rnap2 than with Ctcf in all tissues examined (44.8% vs. 6.7% in liver, 42.5% vs. 14.1% in brain, 72.5% vs. 32.9% in small intestine, 48.5% vs. 1.4% in skeletal muscle) (Figure 3A). These data are consistent with the distinct role of Ctcf in chromatin insulation and enhancer blocking [26], while the shared Rnap2/Rxrα sites may reflect indirect association via binding of Rxrα to sites near expressed genes and direct associations involving the recruitment of Rnap2 to promoter-distal Rxrα-bound enhancer sequences [27,28].

We next performed cross-tissue analyses by determining the proportion of Rnap2, Ctcf and Rxrα binding sites that were shared between tissues (Figure 3B and 3C). Rnap2 and Rxrα binding sites exhibited strong tissue specificity, with between-tissue overlap rates ranging from 48.5% to 58.4% for Rnap2, and 20% to 68.9% for Rxrα (Figure 3B); on average, half of the Rnap2 and Rxrα sites are tissue-specific. In contrast to the tissue specificity of Rnap2 and Rxrα, Ctcf binding was largely similar across tissues, with typical overlap rates exceeding 90% between tissue pairs (Figure 3B). In support of these findings, we also analyzed Rnap2 enrichment at a set of tissue-specific genes and determined that active transcription was limited to the appropriate tissue type (Figure 3C). These observations are consistent with previous studies showing stable Ctcf binding patterns across cell types [29].

We subsequently performed gene ontology analyses for the binding profiles of Rnap2 and Rxrα. Consistently with the colocalization results that pointed to pronounced tissue specificity, genes near Rnap2 and Rxrα binding sites are enriched for distinct biological functions across tissues, and these pathways largely reflect known processes in each tissue (Additional file 2: Figures S2 and S3). For instance, Rnap2 binding sites are enriched for genes related to fatty acid metabolism in liver and muscle cell development in the muscle, while genes near Rxrα binding sites in liver are enriched for lipid and cholesterol metabolic processes, and Rxrα binding in skeletal muscles enriches for genes involved in actin filament-based processes and actin cytoskeleton organization.

We also examined sequence conservation at Rxrα binding sites using genomic evolutionary rate profiling (GERP) scores [30]. In particular, we assessed the difference in conservation between canonical motifs at Rxrα binding sites that are tissue-specific and sites that are bound by Rxrα in two or more tissues (Figure 3D). The data show that, while canonical motifs at Rxrα binding sites are more highly conserved than flanking sequences, the degree of conservation is particularly increased at sites bound in multiple tissues. These data suggest a stronger selective pressure on pleiotropic binding sites relative to tissue-specific sites. In line with the canonical motif data, a similar trend in conservation between tissue-specific and common binding sites is observed when GERP scores are tabulated relative to the binding site peak summit, whether or not a motif is present (Additional file 2: Figure S4).

**Figure 4:**
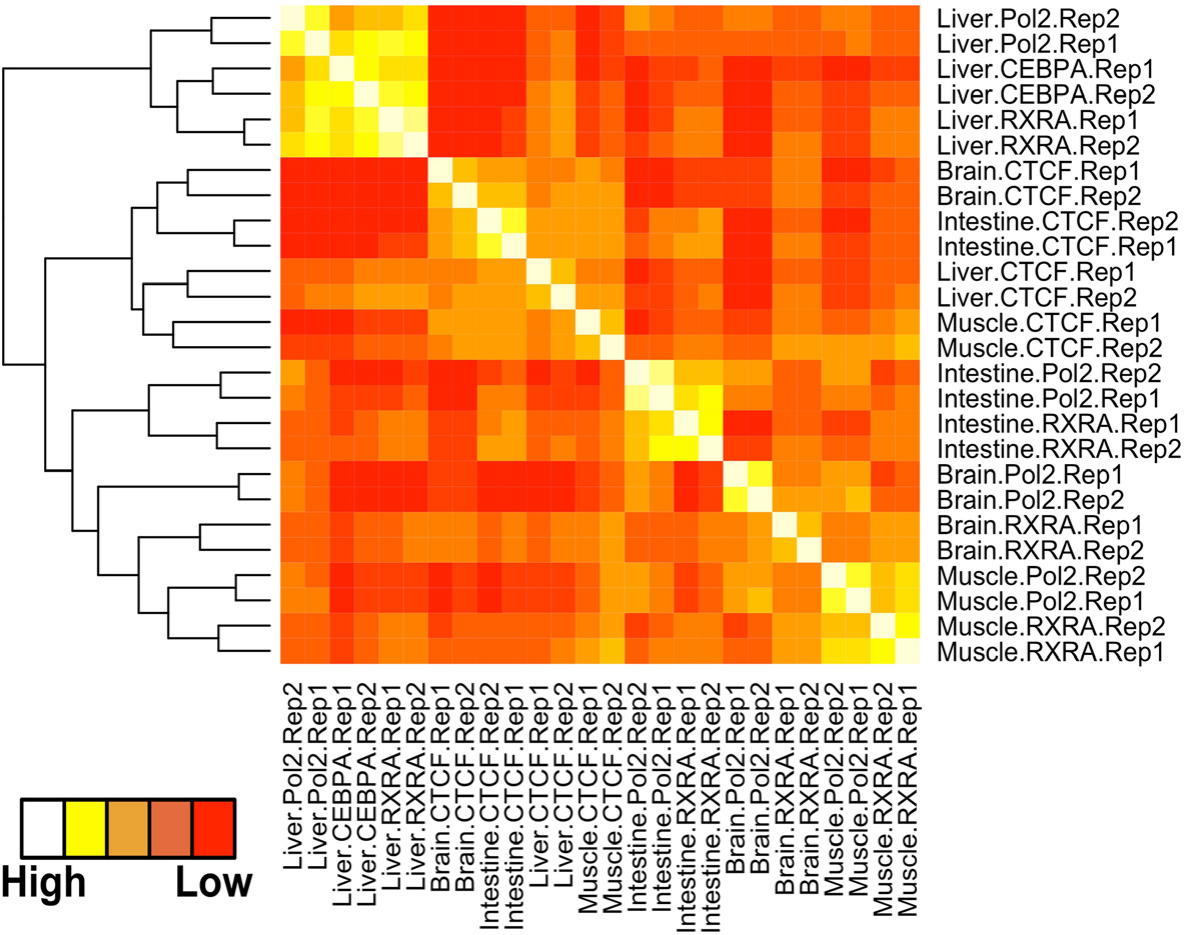
**Correlation matrix between ChIP-seq experiments.** Heat map displaying Spearman rank correlations between all pairwise comparisons for all tissues and ChIPs. Spearman correlations were calculated using the normalized read depth across the entire set of binding sites identified for all ChIP-seq experiments. RNA polymerase II, Pol2; CCCTC-binding factor, Ctcf; Retinoid X receptor α; Rxrα; CCAAT/enhancer-binding protein α, CEBPα.

To define the relationship between each factor across the four tissues that were examined, we looked at the pairwise Spearman rank correlations of normalized read depth within a collective list of binding sites (Figure 4). The resulting correlation matrix captures tissue specificity as the canonical transcription factors (Rxrα and Cebpα) and Rnap2 display a correlation primarily driven by tissue type, while the Ctcf binding patterns form an independent cluster, further supporting the limited cell-type specificity of Ctcf binding [29].

### Replication of results from independent genomic datasets

We used publicly available genomic datasets from mouse tissues, including ChIP-seq, DNaseI hypersensitivity, and RNA-seq to further evaluate how well our data replicate previous analyses. We obtained Rnap2 and Ctcf ChIP-seq data from the Mouse ENCODE Consortium for liver, brain (by combining cortex and cerebellum datasets) and small intestine to assess the fraction of binding sites that are shared [17]. Importantly, these transcription factor binding site datasets were identified using the mincing strategy and therefore allow a direct comparison with the pulverization method. We determined that our ChIP-seq results and mouse ENCODE data exhibited high concordance, as 60 to 75% of Rnap2 binding sites and 78 to 98% of Ctcf binding sites colocalized between datasets in the three tissues examined (Additional file 2: Figure S5). We also evaluated Cebpα binding in liver and determined that more than 90% of our liver Cebpα binding sites (19,155 out of 20,467) were identified in an independent study [25].

Given the lack of available Rxrα ChIP-seq data in murine tissues, we determined the proportion of Rxrα binding sites that coincided with diverse epigenetic modifications identified by the Mouse ENCODE Consortium in liver, brain (by combining cortex and cerebellum data sets) and small intestine [17]. For this analysis, we used histone 3 lysine 4 mono-methylation (H3K4me1) marks, as this modification is found at active chromatin regions and is associated with regulatory elements [31,32], as well as histone 3 lysine 27 tri-methylation (H3K27me3) modifications that are involved in epigenetic silencing [33]. In line with the identification of putatively active *cis*-regulatory enhancer elements, Rxrα binding sites consistently colocalized with active H3K4me1 marks (84.1% in liver, 51.4% in brain, and 87.2% in small intestine), in contrast with H3K27me3 repressive modifications (1.7% in liver, 1% in brain, and 1% in small intestine). Confirming the high ChIP-seq data quality, similar enrichments with histone modifications were also obtained using Rnap2 and Ctcf datasets (Additional file 2: Figure S6).

We also calculated the proportion of Rnap2, Ctcf, and Rxrα binding sites that associated with regions of open chromatin as identified by DNaseI hypersensitivity [34,35] from liver, brain, and skeletal muscle tissues performed by the Mouse ENCODE Consortium. The vast majority of Rnap2 (83.4%), Ctcf (86.8%), and Rxrα (87.2%) sites across these three tissues were situated in regions of open chromatin (Additional file 2: Figure S7). The degree of background or non-specific colocalization was also determined by performing cross-tissue comparisons with ChIP-seq and DNaseI hypersensitivity datasets. We found a significantly stronger enrichment of Rnap2 and Rxrα binding sites with open chromatin regions from the same tissue type compared with cross-tissue comparisons (*P* < 0.05, two-sided Student’s *t* test), while Ctcf binding enrichment with open chromatin was not significantly different between intratissue and intertissue comparisons, further confirming the limited tissue specificity of Ctcf [29].

We also used a publicly available RNA-seq dataset [36] to evaluate the association between Rnap2 ChIP-seq signal and the level of gene expression. For this analysis, we determined the normalized read depth for Rnap2 at gene promoters in liver, brain and skeletal muscle and correlated these values with gene expression measurements (reads per kilobase per million mapped reads or RPKMs) in the same tissue (Additional file 2: Figure S8). Both Rnap2 ChIP-seq replicates from all three tissues exhibited extensive rank correlation with gene RPKMs (0.78 in liver, 0.70 in brain, and 0.77 in muscle), suggesting that our Rnap2 ChIP-seq data at promoters is an accurate predictor of gene expression levels in the same tissue.

### Tissue ChIP-seq input requirements

**Figure 5:**
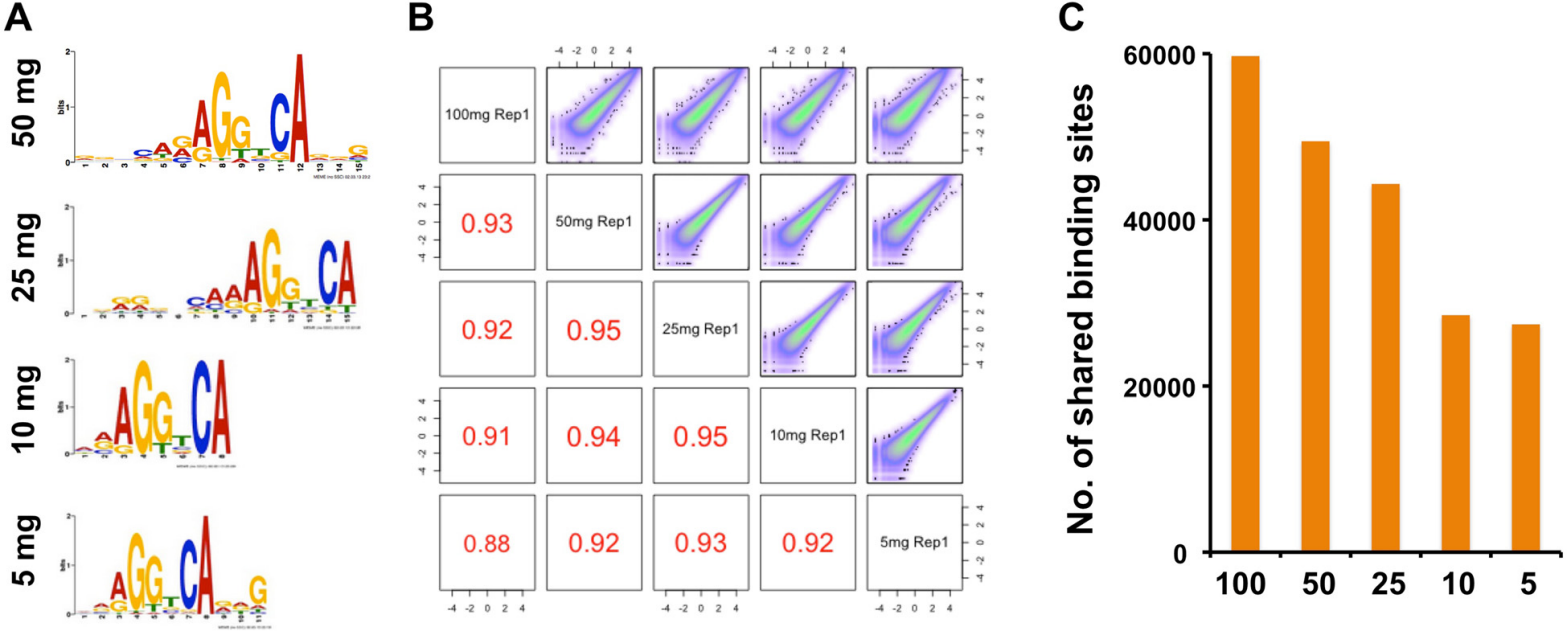
**ChIP-seq liver titration. (A)** Images of the canonical Rxrα motif identified by multiple expectation maximization for motif elicitation (MEME) for all tissue inputs. **(B)** Spearman rank correlations between pairwise comparisons using one replicate from each liver input amount. The upper-right section of the diagram gives the smoothed scatter plot data while the lower-left section displays the rank correlation values, with the font size corresponding to the strength of correlation. **(C)** Bar graph displays the total number of binding sites (*y* axis) shared between biological replicates for each tissue input amount (*x* axis).

To measure the sensitivity of the dry pulverization ChIP method, we performed a titration of mouse liver tissue by performing ChIP-seq targeting Rxrα. For the titration, we sonicated 100 mg fixed liver powder and subsequently performed ChIP-seq on aliquots that corresponded to 50, 25, 10 and 5 mg of tissue. Binding sites that were enriched for the canonical Rxrα binding motif were identified across all tissue input amounts (Figure 5A). Based on previous estimates of hepatocyte number in the mouse liver [37], these data suggest that our assay can generate genome-wide binding profiles using chromatin from as little as ≈675,000 hepatocytes. We determined that each tissue input maintained strong reproducibility, including 92% of binding sites that were shared even between 5 mg biological replicates (Additional file 1: Table S1). Through pairwise comparisons between different tissue amounts, we found that, in each case, more than 90% of binding sites were concordant (Additional file 2: Figure S9). To validate these observations further, we looked at Spearman rank correlations of ChIP-seq signal across tissue amounts and consistently identified strong correlations (Figure 5B). As we anticipated, our data exhibited a positive correlation between the number of binding sites identified and the sample input amount, as increasing amounts of input chromatin lead to a higher number of binding sites (Figure 5C). Despite the change in binding sites, we identified more than 25,000 reproducible Rxrα binding sites with only a small fraction of chromatin that represented 5 mg liver tissue. Remarkably, the assay also retained a high library complexity across all samples (Additional file 1: Table S1). These results suggest that the dry pulverization technique is highly robust and can maximize the number of ChIP-seq experiments performed on limited or rare amounts of frozen tissue samples, such as patient samples.

## Discussion

While many ChIP-seq analyses have been performed in cell lines, primary tissues have not been extensively assayed, even though they may serve as a more appropriate model for evaluating biological function in an organismal context. The labor-intensive and technically challenging nature of previously described protocols involving tissues is likely to have limited the number of primary tissue ChIP-seq investigations. Here we have described and systematically assessed a dry pulverization method for efficiently performing ChIP-seq in frozen mammalian tissues. Importantly, similar pulverization ChIP-seq strategies have been successfully performed in *Drosophila*[38] and *Caenorhabditis elegans*[39] tissues.

We demonstrate that the pulverization strategy generates high-quality data and is highly reproducible, with more than 75% of binding sites being shared across all biological replicates. Our results are also concordant with transcription factor binding site ChIP-seq data obtained using the mincing strategy, as well as DNaseI hypersensitive sites, histone modifications, and gene expression profiles. However, the advantage of the dry pulverization method is in its simplicity, requiring minimal effort and time, thereby making it efficient and adaptable for large-scale projects. Tissue samples can be pulverized in large batches and tissue powder can be stored upon pulverization, prior to crosslinking, allowing laboratories to perform these assays in a higher throughput manner. We also performed all tissue preparations in large batches consisting of several distinct tissue types, and these took only 1 hour to perform. The approach also ensures that tissues remain frozen during the preparation prior to fixation, controlling for potential tissue degradation during sample preparation. The utilization of tissue powder rather than diced tissue for fixation ensures a more homogenous and reproducible fixation, limiting technical variance that may occur through differences in fixation efficiency between tissue replicates or across distinct tissue types. Supporting the advantages of the pulverization method, we determined that the assay was robust, as we successfully performed all ChIP-seq assays by relying on a common set of experimental conditions, despite the different histology and physical characteristics of the tissues examined (see Methods). Collectively, these data suggest that the dry pulverization method captures both meaningful and accurate biological information, yet involves fewer steps and less effort than standard protocols.

We also evaluated the sensitivity of dry pulverization by titrating mouse liver tissue. We demonstrate that the dry pulverization ChIP-seq method is highly sensitive and can identify binding sites from chromatin that corresponds to as little as 5 mg liver tissue, or ≈675,000 hepatocytes, substantially less than previous recommendations for liver tissue [21]. The uniform fixation that occurs through the use of tissue powder may be a contributing factor for the enhanced sensitivity of the pulverization method. In light of these observations, dry pulverization is therefore highly advantageous for handling scarce or limited tissues, such as clinical samples, as it allows for a large number of ChIP-seq experiments to be performed from one sample. However, the lower limit of input amount will probably fluctuate between distinct tissues, as this threshold will be dependent on the nuclear density of a given tissue, in addition to antibody quality. The parameters we used should serve as a starting point for subsequent investigations and additional optimization of conditions may be required for unique tissues not assessed in our study. Although the tissue samples we used for our analyses weighed at least 200 mg prior to pulverization, obtaining powder from smaller initial tissue sample amounts with minimal tissue loss is feasible (see Methods), but downstream sonication and immunoprecipitation conditions may need additional optimization.

The technological advancements over the last decade have allowed for high-resolution studies of genomic processes and these large-scale analyses have led to a deeper understanding of the complexity involved in gene regulation [1,2,9-13]. However, the validation of phenomena observed in cells and the delineation of novel functions in more complex tissues is a critical step for accurately characterizing endogenous biological processes, and for defining genomic anomalies in diseased tissues. We believe that the dry pulverization tissue ChIP-seq approach outlined in this study can facilitate these important next steps.

## Conclusions

We report a dry pulverization method for ChIP-seq analysis of frozen tissues that is robust, reproducible and requires minimal input. The simplicity of dry pulverization and the fact that this technique is amenable to higher throughput analyses collectively makes tissue ChIP-seq analyses widely feasible.

## Methods

### Tissue pulverization and fixation

Tissue samples from C57BL/6J mice at 8 weeks of age were obtained from Jackson Laboratories. Mice were cervically dislocated and tissues were dissected and snap frozen in liquid nitrogen. Tissues were subsequently stored at −80°C until pulverization. Prior to pulverization, a hammer and a metallic block were chilled on dry ice for 20 to 30 minutes. During this preparatory cooling, the metallic block was secured by packing the dry ice around the block to ensure that it would not move during pulverization. Frozen tissue samples (≈200 to 800 mg each) were pulverized in Covaris tissue TUBEs (Covaris 520001) with attached adapters (Covaris 520017) and glass vials (Covaris 520010). To ensure tissues remained cold and brittle during pulverization, Covaris tissueTUBEs were briefly submerged (for ≈5 s) in liquid nitrogen between successive rounds of pulverization (for ≈10 to 20 seconds) using the chilled hammer on the cold metallic block. The tissueTUBE was gently tapped so all tissue matter was collected at the bottom of the bag prior to each pulverization. To ensure that the tissue was fully pulverized, we briefly examined the tissue sample within the transparent tissueTUBE. Typically, complete pulverization takes about 5 to 10 rounds of pulverization, depending on tissue makeup and histology. Once the tissue had been pulverized to a powder, the tissueTUBEs were inverted and tissue powder was collected into attached Covaris glass vials. The subsequent tissue powder was resuspended in room temperature PBS containing protease inhibitors (Roche 11836153001) and transferred to a 15 ml conical tube. Additional room temperature PBS containing protease inhibitors was added to 15 ml conical tubes to a final volume of 10 ml and tissue powder was crosslinked with 1% formaldehyde for 15 minutes while rocking at room temperature. After fixation, the crosslinking reaction was halted using 0.125 M glycine for 5 minutes while rocking at room temperature. Crosslinked tissue powder was pelleted (750 relative centrifugal force for 5 minutes at 4°C) and washed three times with cold (4°C) PBS containing protease inhibitors. After each wash, crosslinked tissue powder was pelleted. Following washing, the pelleted tissue powder was subsequently stored at −80°C. For smaller tissue samples (less than 200 mg), the initial preparation and pulverization should be performed as outlined above. However, the resulting tissue powder should be resuspended with PBS containing protease inhibitors directly within the tissueTUBE, without inverting, to prevent tissue loss. The tissue solution should then be placed in an appropriately sized container and fixed with 1% formaldehyde for 15 minutes.

### Chromatin immunoprecipitation and next-generation sequencing

Antibodies for RNA polymerase II (ab5408, abcam), Rxrα (sc-553, Santa Cruz Biotechnology), Ctcf (sc-5916, Santa Cruz Biotechnology and 61312, Active Motif), and Cebpα (sc-166258, Santa Cruz Biotechnology) were obtained and the ChIP assay and subsequent sequencing library preparations were conducted as previously reported [23]. The Ctcf Active Motif antibody was used for small intestine and the Ctcf sc-5916 antibody was utilized for the remaining three tissues. Sonication was performed with a Sonics Vibracell at 60% amplitude on 100 mg aliquots of fixed tissue powder using six total 30-second durations of sonication for all tissues. All ChIP libraries were run on an Illumina HiSeq 2000 sequencer using 50 bp single-end sequencing.

### Data analysis

Sequencing reads were aligned to the genome using Bowtie [40] and binding sites were identified using the model-based analysis of ChIP-Seq (MACS) peak caller with an mfold cutoff of 15 [41]. All position weight matrices were identified using multiple expectation maximization for motif elicitation (MEME) [42]. Normalized read depths for examining Spearman rank correlations between ChIP-seq experiments were calculated by merging 100-bp binding sites centered on peak summits for all experiments and determining the number of sequencing reads mapping to the entire list of binding sites and normalizing these values by the total number of reads that were mapped for each ChIP experiment (that is, reads per million). For RNA polymerase II promoter analyses, we determined the normalized read depth at a 2-kb region of sequence centered on the transcription start site for all expressed genes detected by RNA-seq. To calculate the fraction of binding sites shared between ChIP-seq experiments, we divided the number of sites that overlapped by the number of sites identified in the smaller ChIP-seq dataset. Evolutionary conservation of motifs was determined by identifying all Rxrα canonical motif position weight matrices within Rxrα-bound sites across all tissues. GERP scores were cataloged for all base positions within the position weight matrix, and binding sites harboring motifs were compared across tissues to determine tissue specificity. For binding site conservation of sites within all peaks regardless of motif presence (Additional file 2: Figure S4), the average GERP scores for each position within a 200-bp fragment of DNA centered on peak summits was used. To determine sequencing library complexity, we calculated the fraction of uniquely mapped reads from a randomly selected sample of 10,000,000 reads that aligned to the mouse genome. Statistical significance was determined with a one- or two-sided Student’s *t* test.

## Abbreviations

Cebpα: CCAAT/enhancer-binding protein α; ChIP: Chromatin immunoprecipitation; ChIP-seq: Chromatin immunoprecipitation next-generation sequencing; Ctcf: CCCTC-binding factor; GERP: Genomic evolutionary rate profiling; MACS: Model-based analysis of ChIP-Seq; MEME: Multiple expectation maximization for motif elicitation; PBS: Phosphate buffered saline; Rnap2: RNA polymerase II; RPKMs: Reads per kilobase per million mapped reads; Rxrα: Retinoid X receptor α.

## Competing interests

The authors declare that they have no competing interests.

## Authors’ contributions

DS participated in designing the study, performing ChIP-seq experimentation, data analysis and in the drafting and revision of the manuscript. JG helped design the study, run ChIP-seq experimentation, and participated in data analysis and in manuscript revision. PJ helped in data analysis and in manuscript revision. GMC aided in data analysis and in revising the manuscript. RMM participated in the coordination, design, and conception of the experimentation, along with manuscript revision. All authors read and approved the final manuscript.

## Supplementary Material

Additional file 1 **Summary of ChIP-seq data.** Table summarizing ChIP-seq experimentation across all mouse tissues. The percentage of sites shared was calculated by dividing the number of shared sites by the number of sites from the biological replicate with a smaller number of identified sites. Click here for file

Additional file 2: Figure S1 Canonical motifs identified in brain, small intestine, and skeletal muscle tissue samples. **Figure S2**. GO biological process analysis for RNA polymerase II ChIP-seq. **Figure S3**. GO biological process analysis for retinoid X receptor ChIP-seq. **Figure S4**. Conservation scores relative to Rxrα binding site summit. **Figure S5**. Analysis of ChIP-seq with Mouse ENCODE transcription factor binding site data. **Figure S6**. Analysis of ChIP-seq with Mouse ENCODE transcription histone modification data. **Figure S7**. Analysis of ChIP-seq with open chromatin annotations. **Figure S8**. Correlation between Rnap2 promoter enrichment and RNA-seq gene expression data. **Figure S9**. Rxrα binding site colocalization across different liver tissue inputs. Click here for file
