# Supplementary material for: Mapping genome-wide transcription factor binding sites in frozen tissues
Source: Epigenetics Chromatin. 2013 Sep 16;6:30. doi: 10.1186/1756-8935-6-30 (PMC3848595; doi:10.1186/1756-8935-6-30)
Supplement: Additional file 1 — Summary of ChIP-seq data. Table summarizing ChIP-seq experimentation across all mouse tissues. The percentage of sites shared was calculated by dividing the number of shared sites by the number of sites from the biological replicate with a smaller number of identified sites. [file 1756-8935-6-30-S1.pdf]

Additional File 1

Table S1: Summary of ChIP-seq data

Table summarizing ChIP-seq experimentation across all mouse tissues. The percent of sites shared (% Shared Sites) was calculated by dividing the number of shared sites by the number of sites from the biological replicate with a smaller number of identified sites.

Tissue	Factor	No. Aligned Reads	Library Complexity	No. Sites	No. Shared Sites	% Shared Sites
Liver	Rnap2 R1	31,107,716	0.97	55190	32853	89%
Liver	Rnap2 R2	19,731,520	0.75	36945		
Liver	Ctcf R1	12,305,618	0.97	10934	9565	87%
Liver	Ctcf R2	15,804,744	0.97	12716		
Liver	Rxra R1	32,674,438	0.91	70740	59716	94%
Liver	Rxra R2	15,184,631	0.87	63414		
Liver	Cebpa R1	15,561,772	0.97	20572	20467	99%
Liver	Cebpa R2	30,191,317	0.94	52740		
Brain	Rnap2 R1	19,495,831	0.97	22232	18569	84%
Brain	Rnap2 R2	22,782,124	0.98	23314		
Brain	Ctcf R1	18,233,756	0.92	23122	18986	82%
Brain	Ctcf R2	13,939,469	0.95	20412		
Brain	Rxra R1	18,517,860	0.97	4663	3559	76%
Brain	Rxra R2	16,843,376	0.97	5585		
S. Intestine	Rnap2 R1	27,881,989	0.94	45172	35975	80%
S. Intestine	Rnap2 R2	27,632,647	0.94	67147		
S. Intestine	Ctcf R1	18,104,474	0.76	54563	49648	91%
S. Intestine	Ctcf R2	18,852,872	0.75	61085		
S. Intestine	Rxra R1	15,805,819	0.98	16901	15615	92%
S. Intestine	Rxra R2	27,445,354	0.98	23416		
S. Muscle	Rnap2 R1	25,637,727	0.97	22334	18967	88%
S. Muscle	Rnap2 R2	18,053,947	0.97	21585		
S. Muscle	Ctcf R1	31,691,471	0.98	15320	2843	99%
S. Muscle	Ctcf R2	20,806,630	0.96	2856		
S. Muscle	Rxra R1	16,265,275	0.94	8169	7993	98%
S. Muscle	Rxra R2	20,877,794	0.96	19130		
Liver	Rnap2-50mg	20,771,903	0.94	55542	49487	90%
Liver	Rnap2-50mg	21,920,417	0.93	55132		
Liver	Rnap2-25mg	21,717,529	0.95	49407	44336	89%
Liver	Rnap2-25mg	19,604,373	0.94	50019		
Liver	Rnap2-10mg	22,629,368	0.96	44176	28553	99%
Liver	Rnap2-10mg	21,144,137	0.97	28819		
Liver	Rnap2-5mg	17,119,872	0.96	32338	27439	92%
Liver	Rnap2-5mg	19,839,578	0.96	29818		
