# Supplementary material for: Mapping genome-wide transcription factor binding sites in frozen tissues
Source: Epigenetics Chromatin. 2013 Sep 16;6:30. doi: 10.1186/1756-8935-6-30 (PMC3848595; doi:10.1186/1756-8935-6-30)
Supplement: Additional file 2: Figure S1 — Canonical motifs identified in brain, small intestine, and skeletal muscle tissue samples. Figure S2. GO biological process analysis for RNA polymerase II ChIP-seq. Figure S3. GO biological process analysis for retinoid X receptor ChIP-seq. Figure S4. Conservation scores relative to Rxrα binding site summit. Figure S5. Analysis of ChIP-seq with Mouse ENCODE transcription factor binding site data. Figure S6. Analysis of ChIP-seq with Mouse ENCODE transcription histone modification data. Figure S7. Analysis of ChIP-seq with open chromatin annotations. Figure S8. Correlation between Rnap2 promoter enrichment and RNA-seq gene expression data. Figure S9. Rxrα binding site colocalization across different liver tissue inputs. [file 1756-8935-6-30-S2.pdf]

Additional File 2

Figure S1: Canonical motifs identified in brain, small intestine and skeletal muscle tissue samples

Rxra and Ctcf motifs identified by MEME from ChIP-seq experiments in mouse brain, small intestine and skeletal muscle is shown.

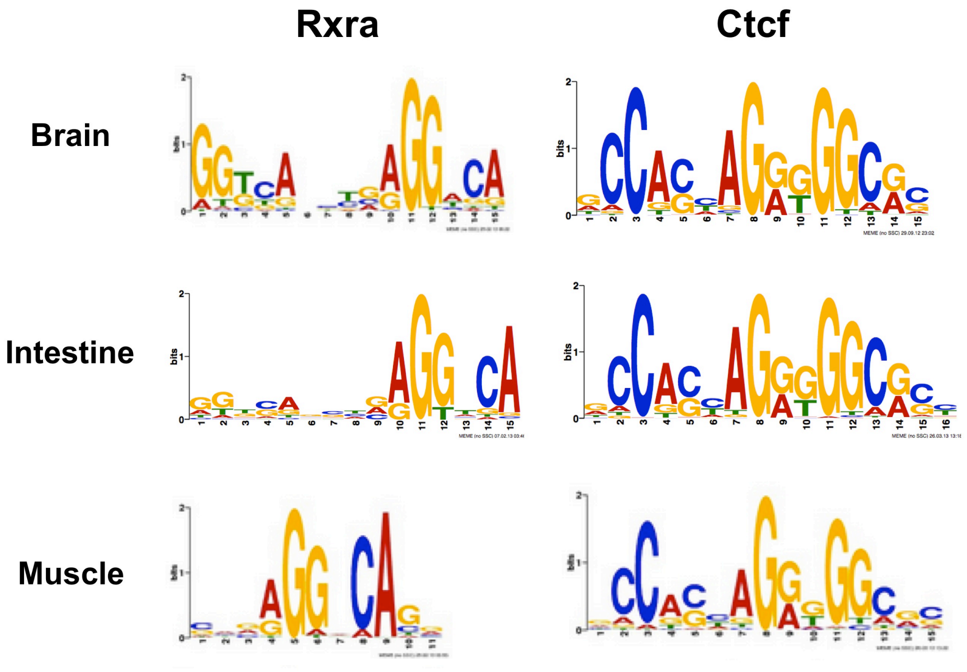

Figure S2: GO Biological Process analysis for RNA Polymerase II ChIP-seq

The top GO Biological Processes for Rnap2 binding sites in distinct tissues is shown.

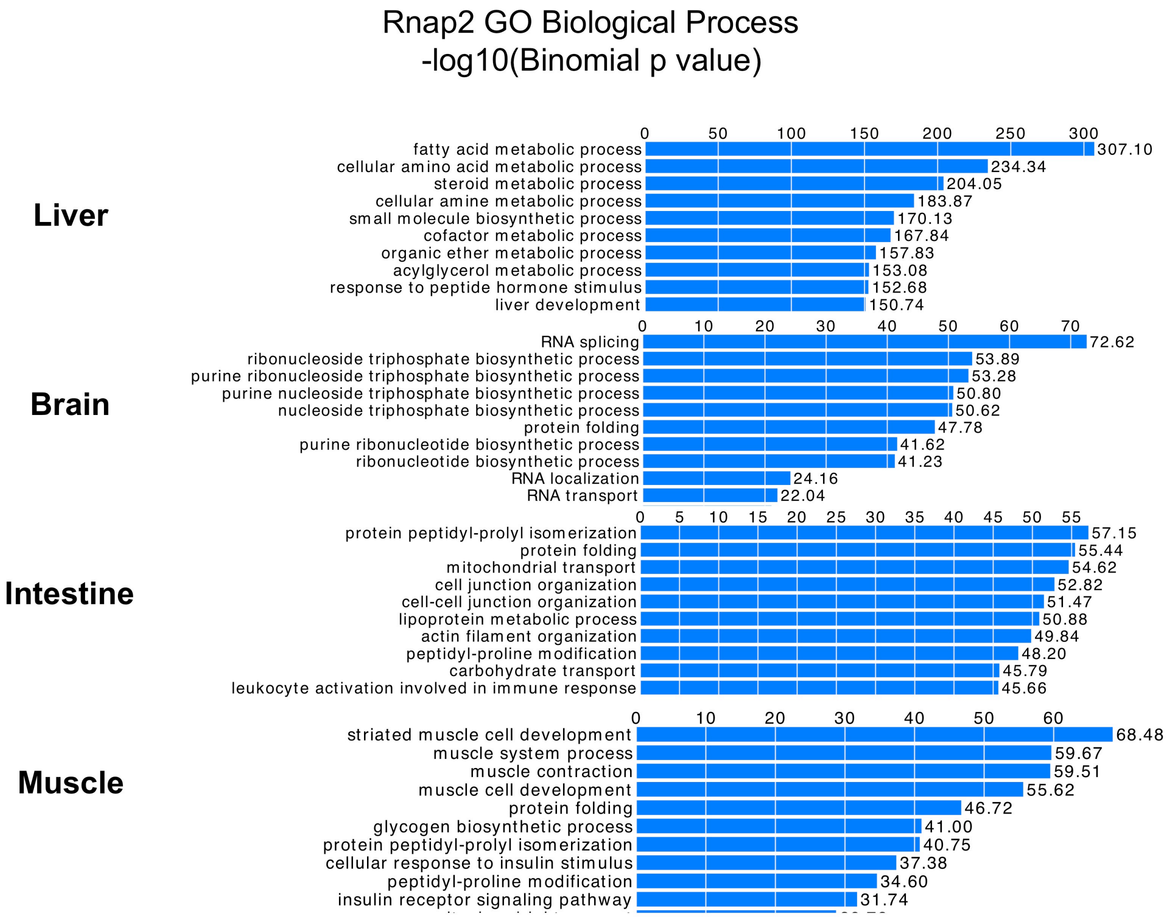

Figure S3: GO Biological Process analysis for Retinoid X receptor ChIP-seq

The top GO Biological Processes for Rxra binding sites in distinct tissues is shown.

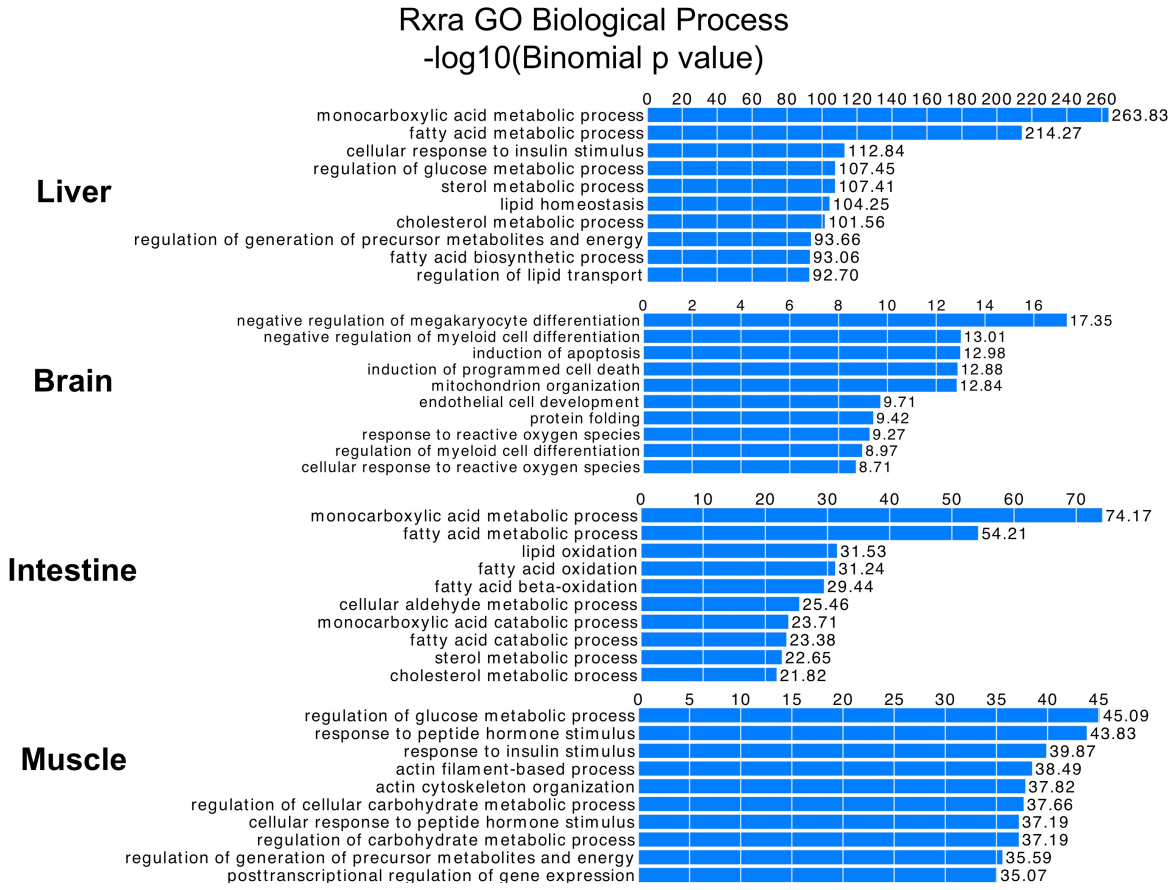

Figure S4: Conservation scores relative to Rxra binding site summit

GERP scores for Rxra tissue-specific sites (dark red) and sites found in two (red) or more (orange) tissues are shown. The y-axis denotes the average GERP score while the x-axis gives the position relative to the summit of the binding site. The plot is smoothed into 5-bp windows. Black dashed line indicates the overall average conservation level for all bases within a 500-bp window centered on the summit positions of the binding sites. There is a highly significant negative relationship between conservation score and distance to summit ($P < 2.2 \times 10^{-16}$, linear regression of GERP score against absolute value of distance to summit), indicative of purifying selection on Rxra binding sites, which tend to be much smaller than, and towards the center of, identified ChIP-seq peaks.

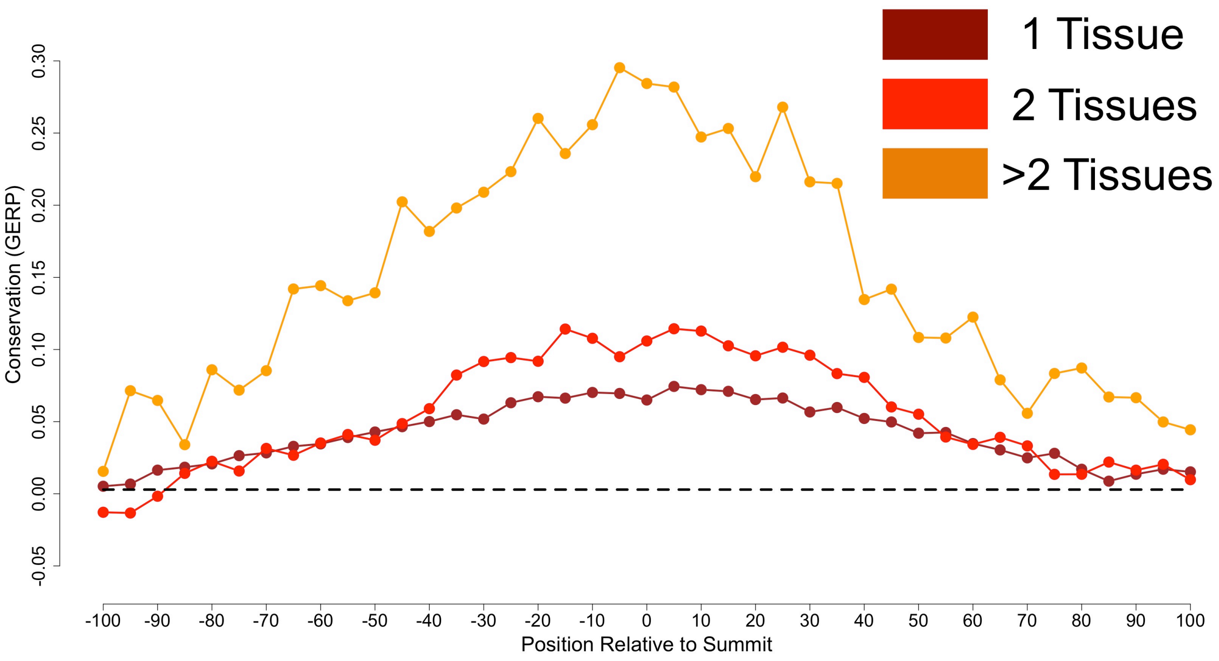

Figure S5: Analysis of ChIP-seq with Mouse ENCODE transcription factor binding site data

Venn diagrams show the overlap (gray) between the dry pulverization ChIPs (light violet) and ChIP-seq data from the Mouse ENCODE consortium (dark violet) for RNA polymerase II (Rnap2) and Ctcf in brain, liver and small intestine. For the ENCODE mouse brain data, the cerebellum and cortex ChIP results were combined. The number of binding sites that co-localized or that were specific to each ChIP experiment are given.

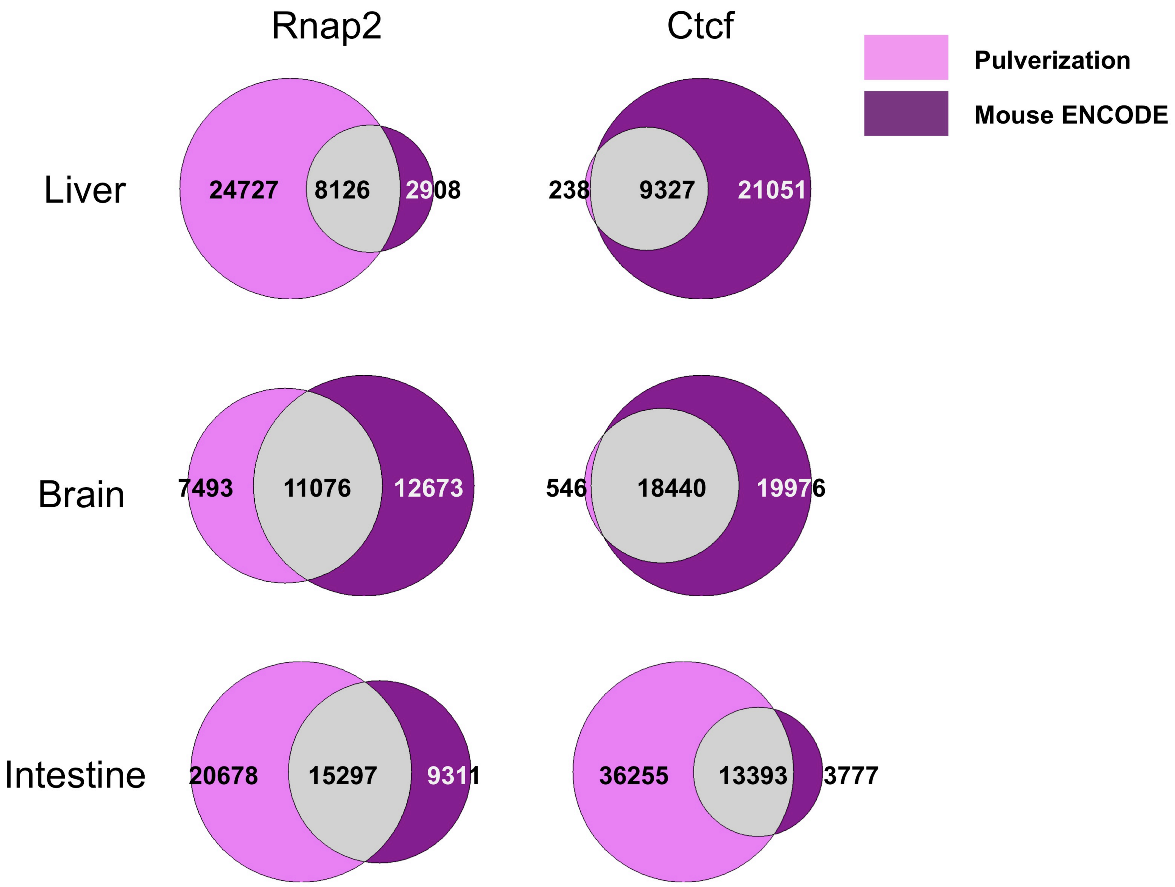

Figure S6: Analysis of ChIP-seq with Mouse ENCODE transcription histone modification data

Bar graphs show the fraction of RNA polymerase II (blue), Rxra (red) and Ctcf (yellow) binding sites that co-localize with the open chromatin mark H3K4me1 and the closed chromatin mark H3K27me3 in liver, brain and small intestine. For the brain H3K4me1 ENCODE data, the cerebellum and cortex ChIP results were combined while only cerebellum data was available for H3K27me3.

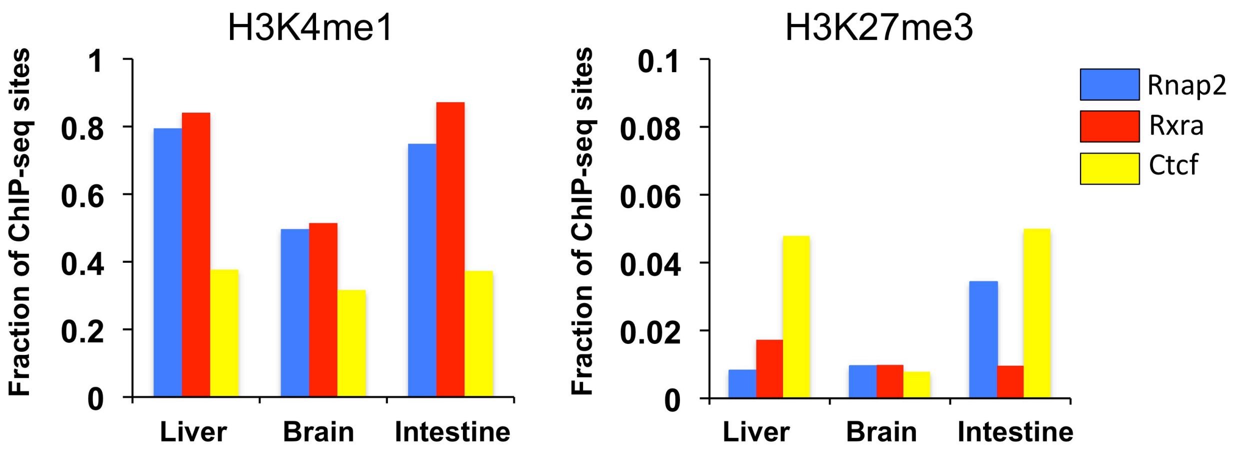

Figure S7: Analysis of ChIP-seq with open chromatin annotations

The fraction of Rnap2 (blue), Rxra (red), and Ctcf (yellow) binding sites that co-localized with regions of open chromatin in liver, brain and skeletal muscles is shown as box plots. Boxes in darker hues give the fraction of Rnap2, Rxra, and Ctcf sites overlapping with regions open chromatin from the same tissue. Boxes in lighter hues (control analyses) display the fraction of ChIP-seq and open chromatin sites that overlap between distinct tissues. *P <0.05.

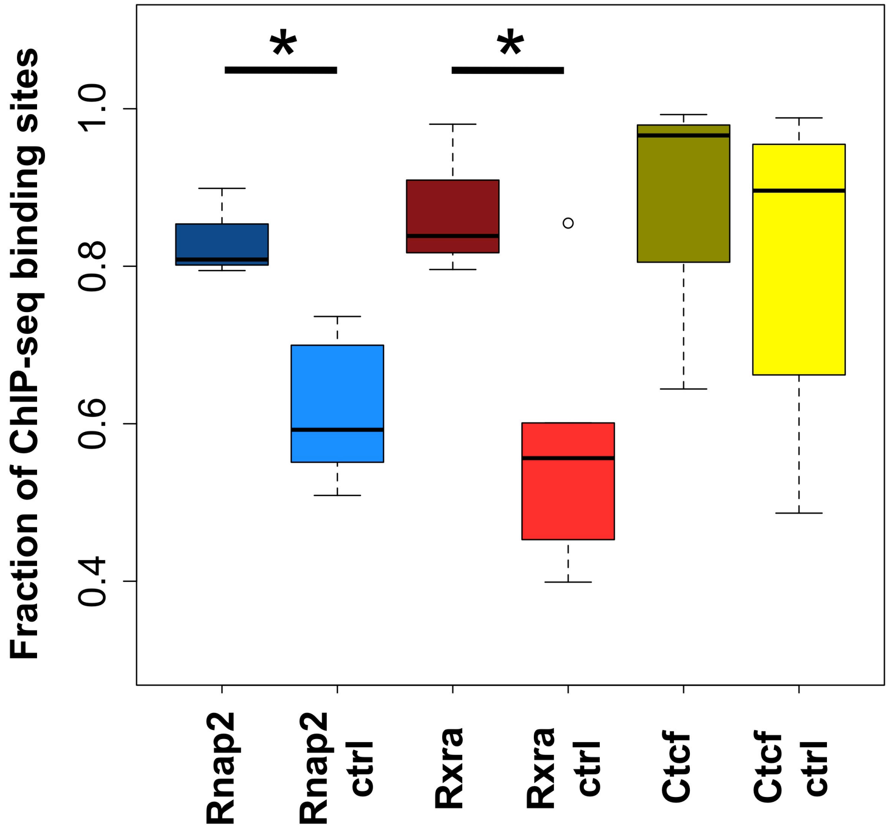

Figure S8: Correlation between Rnap2 promoter enrichment and RNA-seq gene expression data

Scatter plots display the correlation between Rnap2 enrichment at gene promoters and gene expression RPKM values from RNA-seq data across both Rnap2 ChIP-seq replicates in liver, brain and skeletal muscles. Spearman rank correlation values are displayed on each plot.

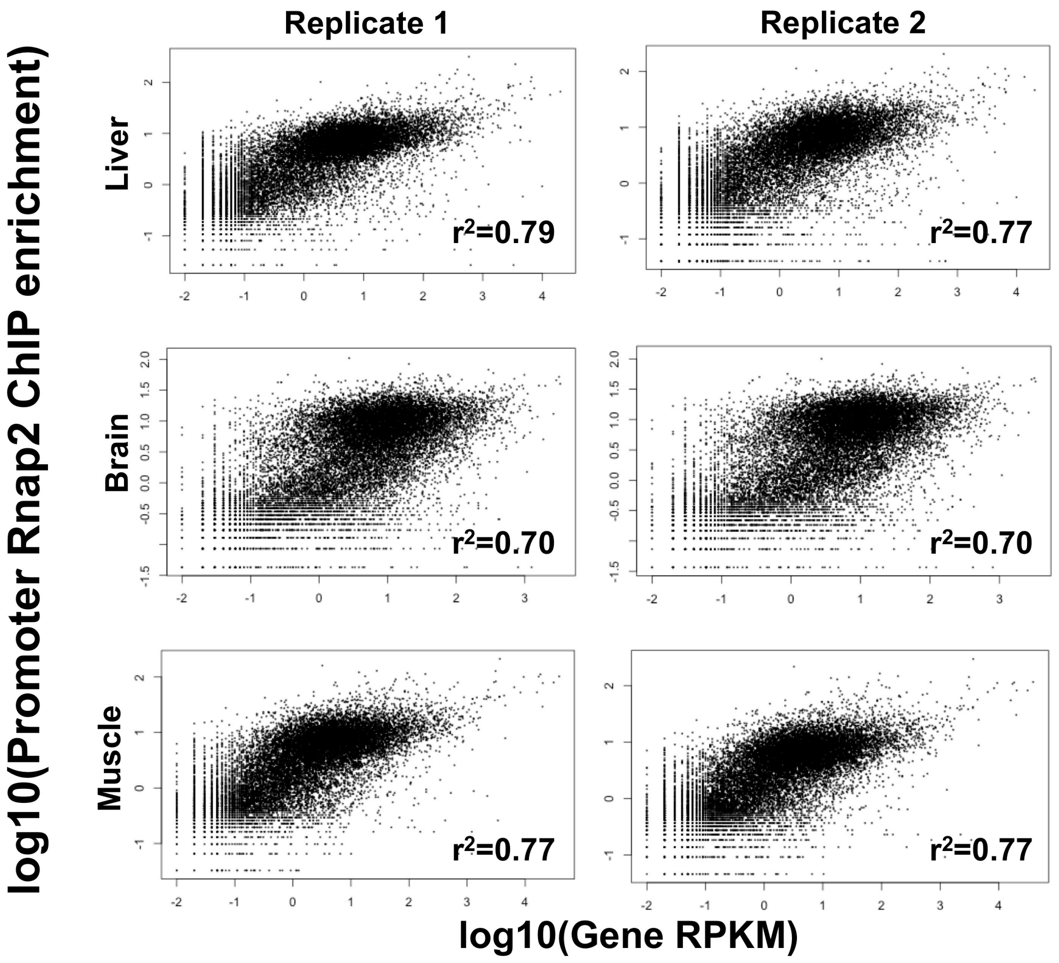

Figure S9: Rxra binding site co-localization across different liver tissue inputs

The total number (top) and percentage (bottom) of binding sites that co-localize across all pairwise tissue input comparisons is shown. For calculating percentages, the number of shared sites was divided by the number of sites identified in the lower tissue input sample.

	100mg	50mg	25mg	10 mg	5 mg
100mg					
50mg	45391				
25mg	41440	42727			
10 mg	27931	28513	28490		
5 mg	26755	27394	27387	24944	

	100mg	50mg	25mg	10 mg	5 mg
100mg					
50mg	91.7%				
25mg	93.5%	96.4%			
10 mg	97.8%	99.9%	99.8%		
